# Study on Lightweight Bridge Crack Detection Algorithm Based on YOLO11

**DOI:** 10.3390/s25113276

**Published:** 2025-05-23

**Authors:** Xuwei Dong, Jiashuo Yuan, Jinpeng Dai

**Affiliations:** 1 Key Laboratory of Opto-Electronic Technology and Intelligent Control, Ministry of Education, Lanzhou Jiaotong University, Lanzhou 730070, China; dxw007@lzjtu.edu.cn (X.D.); 11230852@stu.lzjtu.edu.cn (J.Y.); 2 National and Provincial Joint Engineering Laboratory of Road & Bridge Disaster Prevention and Control, Lanzhou Jiaotong University, Lanzhou 730070, China; 3 School of Materials Science and Engineering, Southeast University, Nanjing 211189, China

**Keywords:** bridge crack detection, YOLO11, lightweight, efficient multiscale convolution, efficient grouped convolution, remote monitoring

## Abstract

Bridge crack detection is a key factor in ensuring the safety and extending the lifespan of bridges. Traditional detection methods often suffer from low efficiency and insufficient accuracy. The development of computer vision has gradually made bridge crack detection methods based on deep learning to become a research hotspot. In this study, a lightweight bridge crack detection algorithm, YOLO11-Bridge Detection (YOLO11-BD), is proposed based on the optimization of the YOLO11 model. This algorithm uses an efficient multiscale conv all (EMSCA) module to enhance channel and spatial attention, thereby strengthening its ability to extract crack features. Additionally, the algorithm improves detection accuracy without increasing the model size. Furthermore, a lightweight detection head (LDH) is introduced to process feature information from different channels using efficient grouped convolutions. It reduces the model’s parameters and computations whilst preserving accuracy, thereby achieving a lightweight model. Experimental results show that compared with the original YOLO11, the YOLO11-BD algorithm improves mAP50 and mAP50-95 on the bridge crack dataset by 3.1% and 4.8%, respectively, whilst significantly reducing GFLOPs by 19.05%. Its frame per second remains higher than 500, demonstrating excellent real-time detection capability and high computational efficiency. The algorithm proposed in this study provides an efficient and flexible solution for the monitoring of bridge cracks using remote sensing devices such as drones, and it has significant practical application value. Its lightweight design ensures strong cross-platform adaptability and provides reliable technical support for intelligent bridge management and maintenance.

## 1. Introduction

Bridge construction in China has been undergoing large-scale and rapid development over the past three decades. By the end of 2024, the number of highway bridges nationwide had reached 1.0793 million, with a total length exceeding 95,288.2 km. Bridges not only play a vital role in carrying traffic but are also crucial in crossing rivers, deep valleys and other obstacles. Thus, they are an essential part of infrastructure construction. Over time, bridges become prone to cracks because of physical and chemical reactions during their service life [1]. Cracks are a common form of structural damage that compromise the integrity of concrete structures [2]. They typically appear when slight damage to the bridge surface occurs. They also lead to weakened and damaged structures through specific structural propagation. Additionally, cracks significantly accelerate the entry of moisture and chemicals into the concrete. As cracks evolve, they can further destabilize the entire bridge structure [3]. Given that cracks accelerate the deterioration process of a bridge, their occurrence and severity are important indicators for determining whether maintenance is required. Crack detection is typically the first step in assessing bridge health and is also a fundamental diagnostic method for evaluating the integrity and lifespan of bridge infrastructure [4]. It helps to predict future conditions and guides predictive maintenance. Ensuring the safety, quality and serviceability of a bridge under limited maintenance resources is possible by detecting cracks early in the bridge’s deterioration process [5].

Bridge crack detection techniques have also been continuously improving and evolving with the development of scientific technologies. For many years, engineers have commonly used visual inspection to detect bridge cracks. However, this method is prone to subjective factors, leading to low efficiency and accuracy, as well as being time-consuming and labor-intensive [6]. Most bridges are built over rivers; thus, challenges and safety risks are present during crack inspection in underwater bridge piers [7]. The development of robotics, which aims to revolutionize traditional detection methods, has been highly anticipated in this field to improve detection capabilities in hazardous areas. At the same time, advancements in computer vision research have made the use of robotic arms and camera-equipped drones a common means of capturing images of various bridge components for crack detection [8]. However, utilizing computer vision technology for bridge crack detection still faces considerable difficulties because of the narrow and elongated shape of bridge cracks, low contrast with the background and interference from significant noise [9]. In previous studies, numerous traditional crack detection methods have been proposed; they primarily include digital image processing techniques (edge detection [10], threshold segmentation [11], region growing methods [12]) and machine learning (support vector machines and random forests) detection methods. Jena et al. proposed a drone-based bridge crack detection model, where the images captured by the drone were processed at an information center to detect bridge cracks [13]. Akagic et al. proposed an unsupervised method to detect cracks on 2D surface images using greyscale histograms and Otsu thresholding [14]. Song et al. developed an image crack detection algorithm based on multiscale pyramids and an improved region-growing method for crack detection [15]. Although digital image processing techniques have improved crack detection levels, they are generally sensitive to background noise interference, thereby reducing crack detection accuracy in complex backgrounds. The widespread use of machine learning has provided an efficient solution for crack detection. Xiao et al. combined an improved region-based active contour model with a linear support vector machine using a greedy search strategy to develop a fully automatic machine learning algorithm for bridge crack detection under various weather conditions [16]. Shi et al. proposed a novel crack detection framework based on a random structure forest, thereby improving detection accuracy and efficiency by applying integral channel features and random structure forests [17]. Although traditional machine learning methods have achieved satisfactory results in some cases, they struggle to extract robust features under varying conditions. Thus, they become unsuitable for crack detection in different environments. In recent years, deep learning technologies have shown excellent performance in crack detection fields. As a branch of machine learning, deep learning can extract high-level abstract features from data by using deep network structures. Thus, it can enhance the model’s crack recognition ability. Object detection is one of the important applications of deep learning. Existing object detection technologies are mainly divided into region-based two-stage detection methods (such as region-based convolutional neural networks [R-CNN], fast R-CNN and Mask R-CNN) and single-stage detection methods using a single network (such as You Only Look Once [YOLO] and Single-Shot MultiBox Detector [SSD]). Xu et al. proposed a concrete bridge crack detection model based on a convolutional neural network. The proposed model outperforms traditional detection models by using dilated convolutions, atrous spatial pyramid pooling (ASPP) modules and depth-separable convolutions. Moreover, this model can be embedded as an effective feature extraction structure in any convolution network [18]. Gan et al. introduced a bridge bottom crack detection method that combines deep learning faster R-CNN and building information modeling to complete crack detection, localization and visualization of beam bridges, which effectively support bridge health assessment [19]. Zhang et al. combined a one-dimensional CNN (1D-CNN) with long short-term memory (LSTM) algorithms to propose a vision-based concrete bridge crack detection method, namely, 1D-CNN-LSTM, which shows higher accuracy and faster computation than existing deep learning methods [20]. Although two-stage algorithms achieve complete end-to-end training and high detection accuracy, their slow computation speed cannot meet real-time requirements. Single-stage object detection algorithms eliminate the two-stage candidate region extraction step, thereby enabling end-to-end object detection with significantly improved detection speed. Compared with two-stage algorithms, the single-stage algorithm avoids the calculation delay of intermediate steps by directly predicting the category and position of objects, thus making object detection more efficient, especially in application scenarios with high real-time requirements, showing great advantages. Zhang et al. proposed an improved YOLOv3-based bridge crack detection algorithm using MobileNets and convolutional block attention modules; the proposed algorithm reduces network parameters to achieve fast and accurate bridge crack detection, which is suitable for timely repair applications [21]. Liu et al. improved the YOLOv5 model by introducing angle regression variables, a new loss function and an attention mechanism module; thus, the detection performance is significantly improved, and the advantages in model size and detection speed are demonstrated [22]. Zou et al. solved the problem of low accuracy in concrete bridge crack detection by using an improved MA-ECA channel attention module to build C2f-MA and adding a small target layer to improve the YOLOv8s model. The improved algorithm demonstrates high accuracy, few parameters and fast speed [23]. Lu et al. proposed an improved SSD that seamlessly integrates depth-separable deformable convolution modules, initial modules and feature recalibration modules to address bridge crack detection challenges [24]. Ruggieri et al. proposed an improved YOLOv11 model of embedded attention mechanism to achieve efficient detection of cracks in reinforced concrete bridges. At the same time, through the Eigen-CAM algorithm, the interpretability of model predictions is enhanced and integrated into the graphical user interface (GUI) to improve the application convenience and practicality in engineering practice [25].

Bridge crack detection requires high real-time performance, and single-stage object detection algorithms have advantages over other methods. As one of the representatives of single-stage object detection algorithms, the YOLO series has continuously improved its performance with version iterations. In October 2024, the Ultralytics team released YOLO11, thereby enhancing feature extraction, optimizing efficiency and speed and further improving detection accuracy. Possessing a few parameters and cross-environment adaptability, YOLO11 is highly suitable for deployment on mobile devices for bridge crack detection tasks. In this study, the YOLO11n model within YOLO11 is optimized to propose a highly suitable YOLO11-BD algorithm for bridge crack detection. The main contributions of this paper can be summarized as follows:

(1) The algorithm uses the efficient multiscale conv all (EMSCA) module to process each feature channel separately, thereby replacing the most information-rich channels and the most relevant spatial regions in the original network structure’s backbone and neck with a C3k2 module. As a result, the bridge crack features are extracted well, and the detection accuracy is improved.
(2) The lightweight detection head (LDH) is used to replace the detection head in the original network, further reducing parameters and floating-point operations (FLOPs) whilst maintaining detection accuracy to achieve model lightweighting.
(3) The improved algorithm has great practical value for the real-time monitoring and maintenance of bridge cracks. It improves detection accuracy and efficiency. Moreover, its lightweight design enables it to be adapted and deployed on various remote sensing devices such as drone and underwater robot, thereby reducing the difficulty of bridge crack detection in complex environments.

## 2. Theory of Algorithm

### 2.1. YOLO11

YOLO, an advanced deep learning object detection algorithm, was first proposed by Joseph Redmon et al. in 2016 [26]. Unlike traditional object detection methods based on sliding windows and region proposals, YOLO regards object detection as a single regression problem. It can complete the recognition and localization of objects in an image through a single glance. It can also simultaneously predict the bounding boxes and class probabilities of multiple categories in a single network. This feature enables it to run at high speeds, thereby making it highly suitable for real-time object detection. The core idea of the YOLO algorithm is to divide the input image into an S × S grid. Each grid cell is responsible for predicting the bounding box and class of objects within that region. In particular, YOLO divides the image into an S × S grid. Each grid cell predicts B bounding boxes, where each bounding box predicts five parameters: the center coordinates (x, y), width (w), height (h) and the confidence score (C), which indicates the presence of an object within the bounding box. The confidence score reflects whether the bounding box contains an object and indicates the accuracy of the predicted box. Additionally, each grid cell predicts the class probabilities for the object. For each bounding box, the algorithm outputs not only the confidence score but also the probability of the bounding box belonging to different object classes. Finally, the YOLO model filters the most appropriate bounding boxes based on these class probabilities and confidence scores. As shown in Equation (1), YOLO uses the intersection over union (*IoU*) to measure the overlap between the predicted box and the ground truth box and to evaluate the prediction accuracy [27].

(1) $$IOU=\frac{Area((bounding\;box)\cap Box(ground\;truth))}{Area((bounding\;box)\cup Box(ground\;truth))}$$

The larger the *IoU* value is, the higher the overlap between the predicted bounding box and the ground truth bounding box is. This scenario indicates high prediction accuracy.

The YOLO algorithm has undergone many versions—from YOLOv1 in 2016 to YOLO11 in 2024. Each new version aims to improve the performance of the previous iteration, thereby further enhancing detection accuracy and speed, whilst reducing error types. A comparison of the main functional modules of the last four versions is shown in Table 1 [28]. YOLO has gone through several developments, thereby achieving significant improvements in accuracy and speed. As a result, it has become the preferred algorithm in fields such as driver-assistance systems, video surveillance and facial recognition; it has also become widely used in civil engineering, agriculture and healthcare [29].

**Figure 1:**
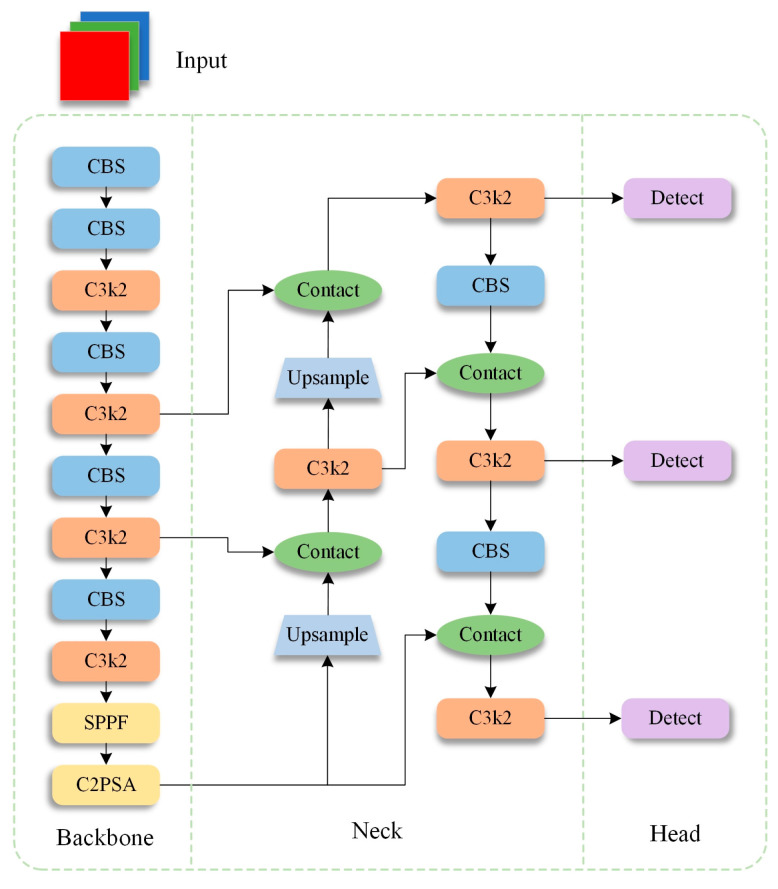
Network structure of the YOLO11.

**Figure 2:**
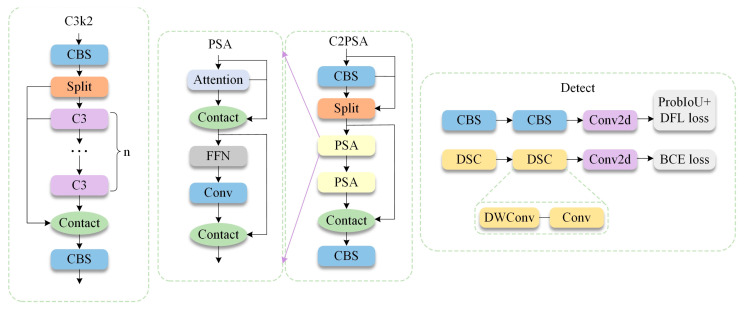
Network optimization module of YOLO11.

The object of this study is YOLO11, a version released by Ultralytics in late 2024. Compared with the previous YOLO versions, YOLO11 introduces new features and improvements, further enhancing its performance and flexibility. Thus, it becomes an ideal choice for various computer vision tasks, such as object detection and tracking, instance segmentation, image classification and pose estimation. The network structure of YOLO11 is shown in Figure 1. It is built upon the previous YOLOv8 version. The improvement of YOLO11 over YOLOv8 mainly lies in the three modules shown in Figure 2, including C3k2, C2PSA and Detect Head. YOLO11 uses a more efficient C3k2 block to replace the C2F module in YOLOv8, providing higher performance without affecting the detection speed. YOLO11 introduces the cross-stage partial with space attention module, which combines the advantages of cross-stage partial networks and self-attention mechanisms. This module enhances spatial attention in feature maps, thereby improving object detection accuracy, particularly for small and overlapping objects [30]. YOLO11 also incorporates the head creativity from YOLOv10 by proposing an anchor-free decoupled head. In particular, the regression branch uses a standard convolutional block, and the classification head uses depthwise separable convolutions (DWConv). Moreover, the binary cross-entropy (BCE) loss is used as the classification loss, whereas distributed feature learning (DFL) loss and complete intersection over union (CIOU) loss are used as regression losses. This approach effectively reduces redundant computations and improves performance [31,32]. Given these improvements, the number of parameters in the YOLO11 model is reduced by approximately 20%.

### 2.2. YOLO11-BD

**Figure 3:**
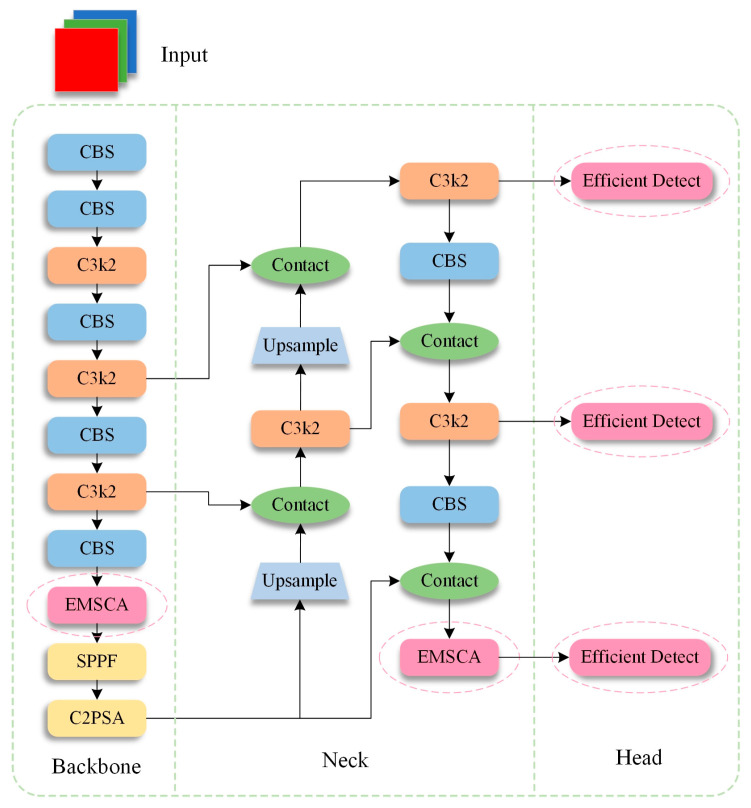
Network structure of the YOLO11-BD.

Although YOLO11 has already been improved based on the previous version, this study proposes an improved algorithm, namely, YOLO11-BD, based on the standard-sized YOLO11n model, to enhance further the accuracy of bridge crack detection and achieve the lightweighting of the model. As shown in Figure 3, in the improved algorithm, efficient multiscale conv all (EMSCA) is used to replace the last C3k2 module of backbone and neck in the original network structure to more fully extract the bridge crack characteristics, reduce parameters and floating-point operations and replace the original detect module with lightweight detection head (LDH) to further reduce the operation complexity while ensuring accuracy. The improvements not only enhance detection accuracy but also significantly reduce the computational load, thereby achieving an increasingly lightweight model.

#### 2.2.1. EMSCA

The efficient multiscale convolution (EMSC) was first introduced and applied to medical image detection and segmentation to enhance the efficiency of CNNs. The core innovation of EMSC is the use of multiscale deep convolution (MSDC) blocks within an attention-based decoding framework to optimize feature map processing and reduce computational costs [33]. EMSC performs multiscale convolution processing on a portion of the input feature map. It also uses depthwise convolutions (DWCs) instead of standard convolutions to operate on each channel independently. Moreover, it utilizes large-kernel grouped attention gates (LGAGs) to refine the feature map by combining it with skip connections from earlier network layers. These improvements help maintain accuracy whilst reducing computation.

**Figure 4:**
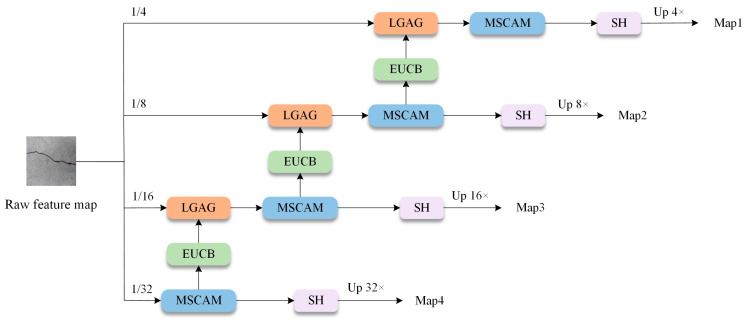
Structure of the EMSCA module.

As shown in Figure 4, this study proposes an improved EMSCA to extract bridge crack features effectively and reduce parameters and FLOPs efficiently. The entire input feature map of the input channels is evenly grouped. Each group of feature maps is processed by the multiscale convolutional attention module (MSCAM). Then, the dimensions and resolutions of the feature maps in the next stage are matched through the efficient up-convolution block (EUCB). Afterwards, LGAG is used to fuse the results from all multiscale convolutions across channels. Then, a 1 × 1 convolution (SH) and upsampling are conducted to enhance the feature map output.

Unlike traditional attention mechanisms (such as SE, CBAM), EMSCA combines multiple innovative modules. For example, EMSCA combines MSCAM with LGAG, effectively captures feature information of different scales by using multiscale features and enhances the expressive ability of features through multi-level operations. In contrast, CBAM and SE modules usually only perform simple weighting operations on the channel dimension or spatial dimension, while EMSCA combines different convolution and pooling methods to enable the model to learn feature on multiple scales, thereby improving the adaptability to complex scenarios.

This study applies EMSCA to capture local contextual information in the most informative channels and the most relevant spatial regions and to improve the channel attention and spatial attention of MSCAM. As a result, the last C3k2 module with the largest number of channels in the backbone and neck is replaced.

**Figure 5:**
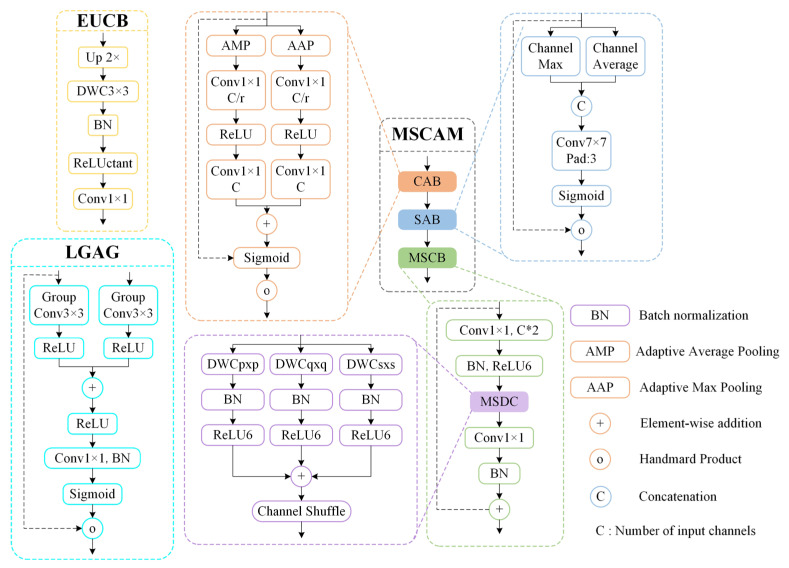
Core modules of EMSCA.

Figure 5 shows the three core modules of EMSCA: *MSCAM*, EUCB and LGAG. *MSCAM* consists of a channel attention block (*CAB*) to enhance channel relationships, a spatial attention block (*SAB*) to capture local contextual information and an EMSC block (*MSCB*). Compared with convolutional attention modules, *MSCAM* significantly reduces computational costs and improves efficiency by applying deep convolutions at multiple scales [34]. The relationships amongst the three modules are expressed in Equation (2).

(2) MSCAM(x)=MSCB(SAB(CAB(x)))

*MSCB* is an EMSC block designed to enhance features generated by the cascade expansion path. Firstly, *MSCB* extends the number of channels using a 1 × 1 convolution (*PWC*_1_). This approach is followed by batch normalization (*BN*) and ReLU6 activation. Then, MSCB uses MSDC to capture multiscale and multiresolution contexts. Finally, the original channel count is restored using another 1 × 1 convolution (*PWC*_2_) and BN. The formula for *MSCB* is defined in Equation (3).

(3) $$MSCB(x)=BN(PWC_{2}(CS(MSDC(R6(BN(PWC_{1}(x)))))))$$

*CAB* assigns different levels of importance to each channel. Thus, the performance can be optimized by emphasizing relevant features and suppressing less useful features. *CAB* applies adaptive max pooling (Pm) and adaptive average pooling (Pa) along the spatial dimension, followed by a 1 × 1 convolution (C_1_) and ReLU activation (R) to reduce the number of channels to 1/16 of the original. Then, it restores the channel count using another 1 × 1 convolution (C_2_) and estimates attention weights with a sigmoid activation before merging these weights into the input x using the Hadamard product (o). The formula for *CAB* is defined in Equation (4).

(4) $$CAB(x)=\sigma (C_{2}(R(C_{1}(P_{m}(x))))+C_{2}(R(C_{1}(P_{a}(x)))))\circ x$$

*SAB* enhances the model’s ability to recognize and respond to relevant spatial features by focusing on specific parts of the input image and mimicking the human brain’s attention mechanism. *SAB* pools the max (Chmax) and average (Chavg) values along the channel dimension to focus on local features. Then, a large kernel (7 × 7) convolution is used to strengthen the local context relationships between features. *SAB* applies a sigmoid activation to calculate attention weights. These weights are assigned to the input x. The formula for *SAB* is defined in Equation (5).

(5) $$SAB(x)=\sigma (LK(C([Ch_{\max}(x),Ch_{avg}(x)]))\circ x)$$

*EUCB* module gradually upsamples the feature map of the current stage through an *EUCB* to match the size and resolution of the feature map of the next skip connection. The module uses an upsampling operation (U_P_) with a magnification factor of two to enlarge the feature map. Then, it enhances the enlarged feature map by applying a 3 × 3 *DWC*, *BN* and *ReLU* activation. Finally, it reduces the number of channels using a 1 × 1 convolution (*C*_1×1_) to match the next stage. The formula for *EUCB* is defined in Equation (6).

(6) EUCB(x)=C1×1(ReLU(BN(DWC(Up(x)))))

*LGAG* combines feature maps with attention coefficients, thereby allowing the network to learn these coefficients to activate relevant features strongly, whilst suppressing irrelevant features. This process uses gating signals derived from high-level features to control the flow of information through different stages of the network, thereby improving precision. *LGAG* applies separate 3 × 3 grouped convolutions (*GC_g_* and *GC_x_*) to process g and x. It also uses *BN* to normalize these convolutional features and merges them using element-wise addition. The resulting feature map is activated through a ReLU layer. Then, it is passed through a 1 × 1 convolution (*C*) and *BN* to obtain a single-channel feature map [35,36]. These feature maps are passed through a sigmoid activation to generate attention coefficients, which are then used to scale the input feature x through element-wise multiplication. As a result, the attention-gated feature *LGAG* (g, x) is produced. The formula for *LGAG* is defined in Equations (7) and (8).

(7) $$q_{att}(g,x)=R(BN(GC_{g}(g))+BN(GC_{x}(x)))$$

(8) $$LGAG(g,x)=x\circ \sigma (BN(C(q_{att}(g,x))))$$

#### 2.2.2. LDH

In bridge crack detection, drones are gaining increasing attention because of their high mobility. Therefore, the lightweight crack detection models that can be carried by drones have become a key research area of detection algorithms. Lightweight models reduce the load on drones, extend flight time and improve data processing speed. Moreover, they enable real-time monitoring and analysis. LDH is used to meet this lightweight requirement. This detection head reduces computational resource consumption by adopting an efficient convolutional network structure, whilst ensuring detection accuracy and speed. This structure allows the model to perform quickly and effectively even on drones with limited computational capabilities, thereby enabling precise identification of bridge cracks.

**Figure 6:**
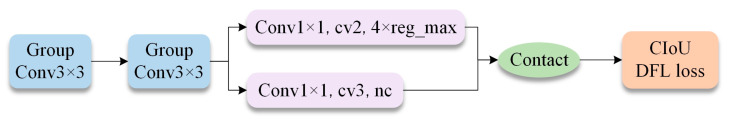
Structure of LDH.

The core advantage of LDH lies in its fine feature extraction capability and optimized convolution structure. The detection head can independently process information in different channel groups by using grouped convolutions, thereby accurately capturing key details, such as small cracks. Figure 6 shows that the introduction of grouped convolutions further enhances the learning of local features, which is particularly critical for improving model recognition accuracy for complex or small targets. LDH also integrates DFL and advanced regression techniques (CIOU), thereby providing precise location predictions and class probability calculations. DFL enhances spatial precision for predicted bounding boxes, whereas CIOU optimizes the alignment between bounding boxes and real targets, significantly improving detection accuracy. Additionally, LDH features dynamic grid reconstruction and adaptive export modes, thereby allowing the model to adjust its structure dynamically in response to real-time data. As a result, its response is optimized for different input variations. This flexibility makes the model suitable for various monitoring environments, thereby greatly enhancing its reliability and efficiency in practical applications.

## 3. Dataset

**Figure 7:**
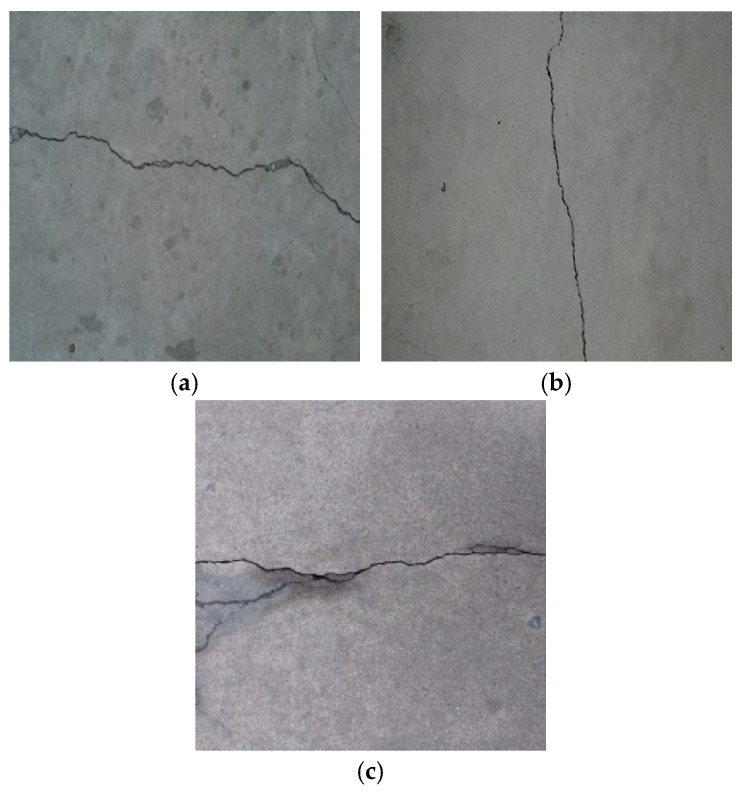
Bridge cracks of different shapes. (**a**) Horizontal crack. (**b**) Vertical crack. (**c**) Crocodile crack.

**Figure 8:**
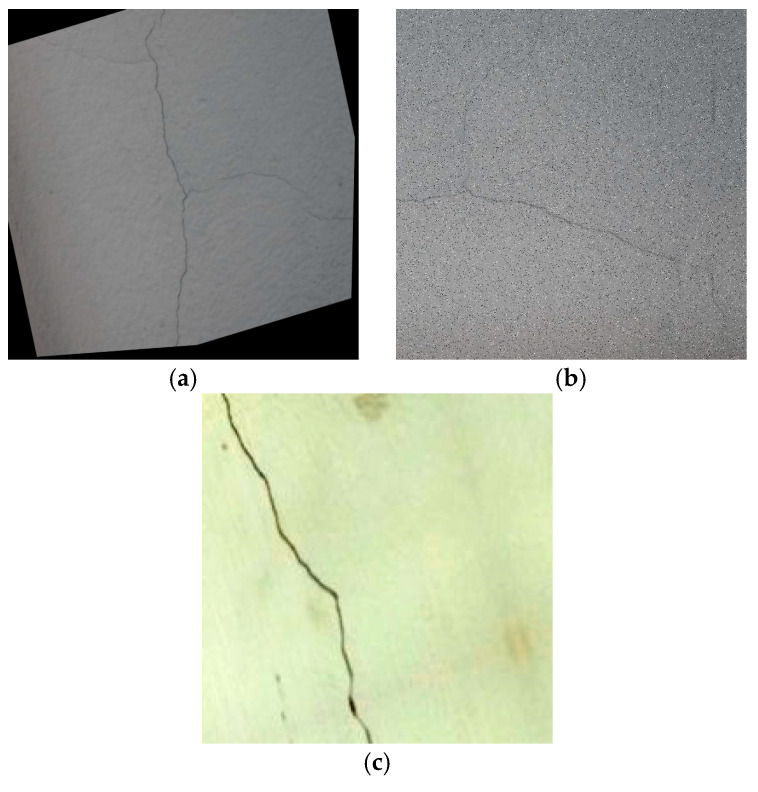
Augmented images with data enhancement processing. (**a**) Rotate. (**b**) Increase noise. (**c**) Underwater.

The experiment collected 500 bridge crack images from the Roboflow dataset website recommended by YOLO to validate the performance of the improved algorithm in bridge crack detection. As shown in Figure 7, the selected image comes from multiple different bridges, covering cracks in the form of lateral cracks, longitudinal cracks, and crocodile cracks. The cracks have different widths and lengths, covering concrete surfaces with different materials and under various lighting conditions. Given the complexity of bridge environments, such as underwater pier cracks, the dataset was augmented to simulate various crack scenarios, thereby improving the model’s generalization ability. Figure 8 shows that the images in the dataset were rotated and cropped. Moreover, noise was added, and color transformations were applied (to simulate different water environments). After data augmentation, the dataset is expanded to 2104, with the total number of labelled bounding boxes being 2873, and divided into training sets, validation sets and test sets, in the ratio of 8:1:1.

## 4. Experimental Environment

The programming language used in this experiment is Python 3.12, with the open-source deep learning framework PyTorch 2.3.0 being the network framework. Cuda 12.1 is used to accelerate the model training speed. The hardware environment includes a CPU (16 vCPU Intel Xeon Platinum 8352V CPU @ 2.10 GHz, Inter Corporation, made in Malaysia) and a GPU (RTX 4090 [24 GB] × 1, NVIDIA Corporation, made in Tawain).

During training, the image size is set to 640 × 640 pixels. The training process includes 200 epochs, with each batch exhibiting a batch size of 128. Data loading utilizes 16 worker threads to accelerate processing. Early stopping is enabled if the training performance does not improve after 50 iterations. The training uses the SGD optimizer, and mosaic data augmentation is turned off during the last 10 epochs to avoid excessive data disturbance at the end of the training. All training processes are conducted on the GPU device. Given that this study focuses on the improvement of EMSCA and LDH on the YOLO11 architecture, in order to ensure the consistency of experimental results comparison, all hyperparameters (such as learning rate, momentum, etc.) are set by default, and no adjustments are made.

All experiments for different algorithms are conducted under the same hardware and software environment to ensure the fairness and comparability of the experiments. The training parameters and dataset settings for each model remain consistent. This scenario ensures that external factors have a minimal impact on the results, thereby making the performance comparison between models highly accurate and convincing.

## 5. Evaluation Index

This study uses four evaluation indicators, namely mean average precision (*mAP*) (including mAP50 and mAP50-95), F1, giga floating-point operations per seconds (GFLOPs) and frame per second (FPS) to evaluate the algorithms in the experiments. In the performance evaluation of object detection, mAP50 and mAP50-95 are key indicators, which have high credibility and practicability. MAP50 represents the average precision when the *IoU* threshold is 0.5; mAP50-95 is the average precision calculated across multiple *IoU* thresholds ranging from 0.5 to 0.95, providing a strict assessment of the model’s detection ability. F1 is a combined metric of precision and recall, which helps avoid extreme situations where precision and recall differ significantly. It also reflects the overall performance accurately. Both mAP and F1 are calculated based on precision (*P_r_*) and recall (*R_e_*). Precision represents the proportion of the actual positive samples in the predicted samples out of all positive samples, whereas recall represents the proportion of actual positive samples amongst all predicted samples. GFLOPs is an important metric for measuring the computational complexity of object detection algorithms. It represents the number of FLOPs performed per second (in billions). The value of GFLOPs directly affects the training and inference speed of the model, particularly when hardware resources are limited. Reducing GFLOPs is a key to improving model efficiency. FPS is an indicator of the inference speed of an object detection algorithm. It also represents the number of image frames that can be processed per second. The higher the FPS is, the stronger the model’s responsiveness in real-time applications is. Thus, the model can handle additional data. The formulas for calculating the above indicators are as follows (Equations (9)–(12)).

(9) $$P_{r}=\frac{T_{P}}{T_{P}+F_{P}}$$

(10) $$R_{e}=\frac{T_{P}}{T_{P}+F_{N}}$$

(11) $$F_{1}=2\cdot \frac{P_{r}\cdot R_{e}}{P_{r}+R_{e}}$$

(12) $$mAP=\frac{1}{n}\sum_{i=1}^{n}\int_{0}^{1}P_{r}\left(R_{e}\right)d\left(R_{e}\right)$$

where *T_P_* represents true positives (the number of correctly predicted crack samples), *F_P_* represents false positives (the number of non-crack objects predicted as cracks), *F_N_* represents false negatives (the number of crack objects that were not detected), and n represents the number of categories in object detection.

## 6. Experimental Results

### 6.1. Ablation Experiment Comparison

An ablation experiment was conducted to verify fully the impact of each component of the proposed model on the prediction performance. The experimental results are shown in Table 2. When the EMSCA module was introduced into the YOLO11 algorithm, both mAP50 and mAP50-95 were significantly improved, while GFLOPs remained unchanged. Although the improvement in mAP50 and mAP50-95 is slightly inferior to that of EMSCA module after the introduction of the LDH module, GFLOPs has been greatly reduced. However, when the YOLO11n-BD algorithm introduced these two improvement modules simultaneously, all indicators showed significant optimization effects. The F1 score of YOLO11n-BD reached 89.2%, a notable improvement over YOLO11n’s 86.8%. This finding indicates that the accuracy and robustness of YOLO11n-BD in crack detection are stronger than those of YOLO11n. In terms of mAP50 and mAP50-95, YOLO11n-BD achieved 94.3% and 71.4%, respectively, showing improvements of 3.1% and 4.8% over YOLO11n’s mAP50 and mAP50-95 values. This finding highlights YOLO11n-BD’s enhanced multiscale crack detection ability. Moreover, the GFLOPs of YOLO11n-BD decreased to 5.1, a substantial reduction of 19.05% compared with those of YOLO11n. This observation demonstrates the model’s lightweighting effect, whilst maintaining high accuracy and reducing computational load. Moreover, YOLO11n-BD maintained 555 FPS, which was slightly higher than the FPS of YOLO11n. This finding shows YOLO11n-BD’s advantage in real-time performance. Overall, YOLO11n-BD demonstrates excellent application potential in bridge crack detection, thereby meeting the demand for efficient detection in complex environments through accuracy improvement, lightweight design and real-time performance.

**Figure 9:**
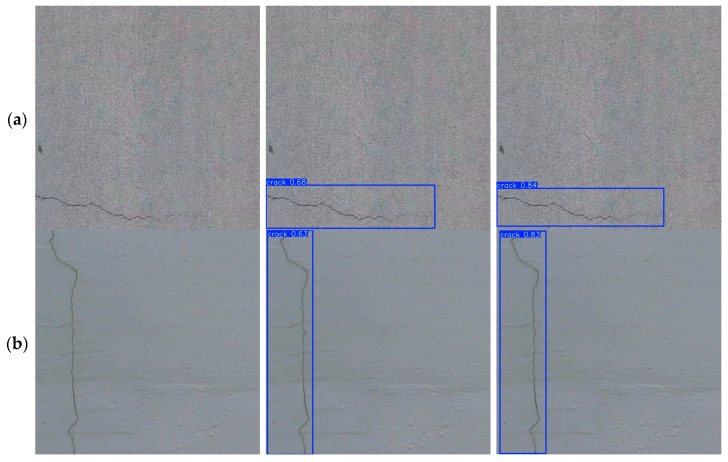

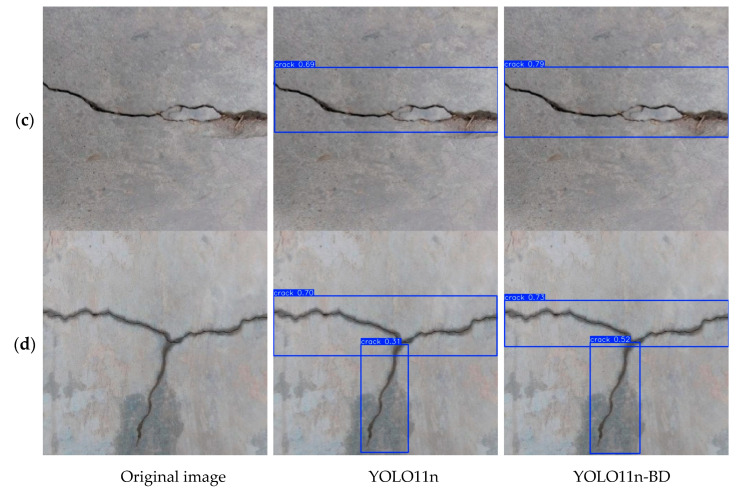
Comparison of detection effects in ablation experiments.

Figure 9a–d shows the crack detection results of YOLO11n and YOLO11n-BD algorithms on the Roboflow dataset. The figures show that compared with YOLO11n, YOLO11n-BD shows an obvious improvement in accuracy and detail capture for crack detection. In detecting the subtle parts of the crack, YOLO11n-BD can precisely define the crack edges, thereby reducing misidentifications. This improvement is achieved by enhancing the algorithm’s ability to recognize crack features, thereby enabling the model to effectively distinguish cracks from non-crack elements in complex backgrounds. As a result, the overall detection performance and reliability are improved.

### 6.2. Comparison of Detection Performance with Different Algorithms

Mask-RCNN and YOLOv10 were selected for comparison in the experiment to validate further the effectiveness and efficiency of the YOLO11-BD algorithm. The experimental results are shown in Table 3. The comparison with different algorithms indicated that YOLO11-BD demonstrates significant advantages over Mask-RCNN, YOLOv10 and YOLO11 in terms of detection accuracy, computational load and operational efficiency. Firstly, YOLO11-BD achieves significant improvements in mAP50 and mAP50-95, which are increased by 9.1% and 11.1%, respectively, compared with those of Mask-RCNN. Secondly, although YOLO11-BD achieves accuracy improvements, the GFLOPs have significantly decreased, and FPS has improved.

**Figure 10:**
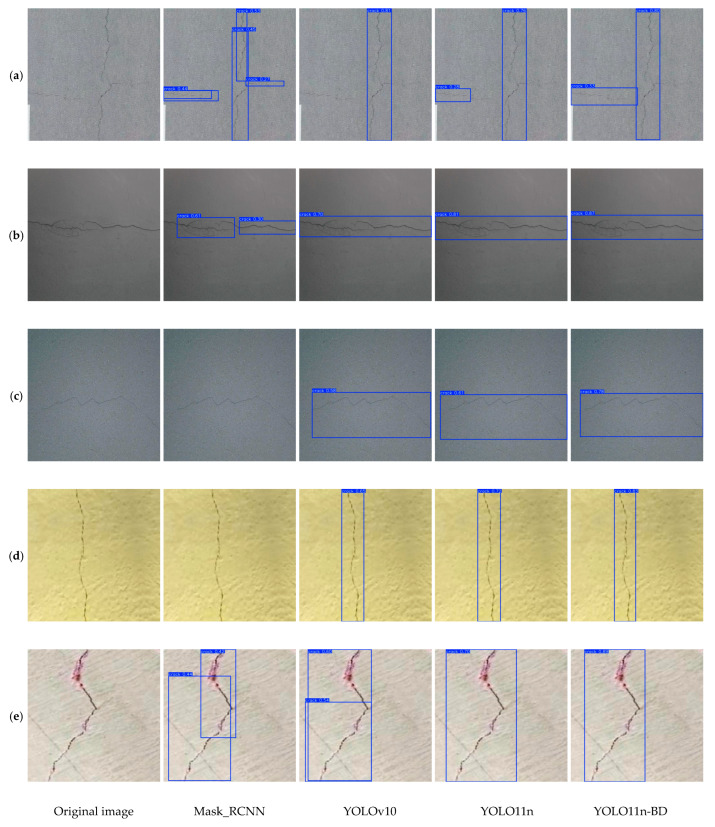
Comparison of detection effects of different algorithms.

The results show that the algorithm not only maintains a high FPS but also optimizes detection performance and computational efficiency. Thus, it requires few hardware resources and enables a highly efficient operation. It is particularly suitable for devices with limited resources or scenarios requiring quick responses, thereby providing better performance in practical applications. In addition, the comparison of the detection results in Figure 10a–e shows that YOLO11-BD can detect cracks accurately and perform precise bounding box selection. Its annotation of cracks shows a high detection confidence level and highly accurate bounding box results. The Mask-RCNN algorithm shows missed detections when dealing with images with noise interference and in different background environments, such as the two groups of Figure 10c,d. In comparison, YOLO11-BD can maintain a high detection effect and overcome the interference caused by the color difference of the background. This observation indicates that YOLO11-BD achieves a good balance between accuracy and efficiency. Thus, it is suitable for application scenarios that require high precision and fast response.

In the previous experiments, in order to accelerate the training speed of the model, a high-performance GPU with RTX 4090 was used. To further assess the model’s adaptability and deployment performance in different hardware platforms, a relatively lower-performing RTX 4060 GPU is selected to evaluate the training performance under the same configuration. The goal is to examine the effectiveness of the YOLO11-BD algorithm in terms of inference speed (FPS) and performance metrics. The experimental results are presented in Table 4.

The results show that compared with the RTX 4090 environment, the YOLO11-BD algorithm still shows excellent accuracy and computing efficiency in the RTX 4060 environment. Although the FPS has decreased due to the differences in GPU hardware, the overall performance is still excellent, demonstrating the universality of the YOLO11-BD algorithm to deploy and apply on resource-limited devices.

### 6.3. Cross-Validation

In order to verify the robustness of the proposed YOLO11-BD algorithm under different dataset division conditions, this paper adopts five-fold cross-validation as the performance verification method. Specifically, the complete dataset is divided into five subsets of similar sizes which do not overlap. In each round, one of the subsets is selected as the validation set, and the remaining four subsets are used for model training, and the loop is performed five times to ensure that each subset is used as the validation set once. This method effectively reduces performance fluctuations caused by random division, thereby improving the statistical credibility of the results. During the cross-validation process, the model architecture, training strategies and all hyperparameters are consistent to ensure the consistency and fairness of the experiment. The results of five-fold cross-validation are shown in Table 5.

After five-fold cross-validation, the average F1 of the model was 89.2%, the average mAP50 was 94.5% and the average mAP50-95 was 71.6%. These experimental results show that YOLO11-BD always maintains excellent detection performance in multiple rounds of verification, which fully reflects the robustness and generalization ability of the proposed improvement method in actual scenarios.

### 6.4. Detection in Different Complex Scenarios

**Figure 11:**
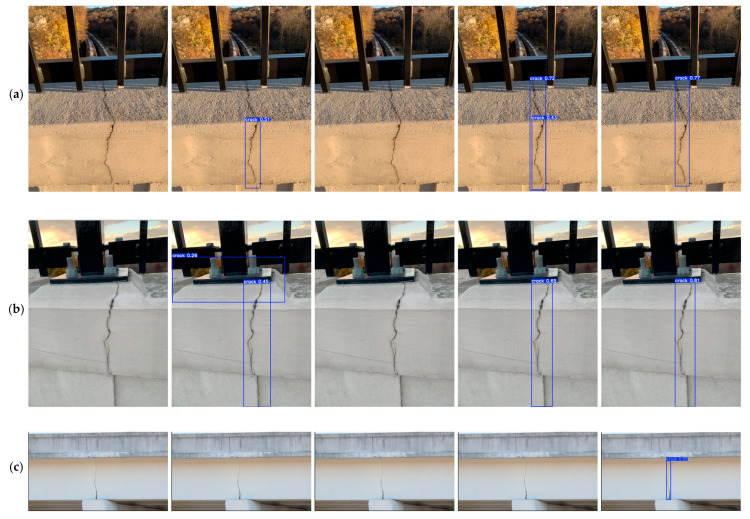

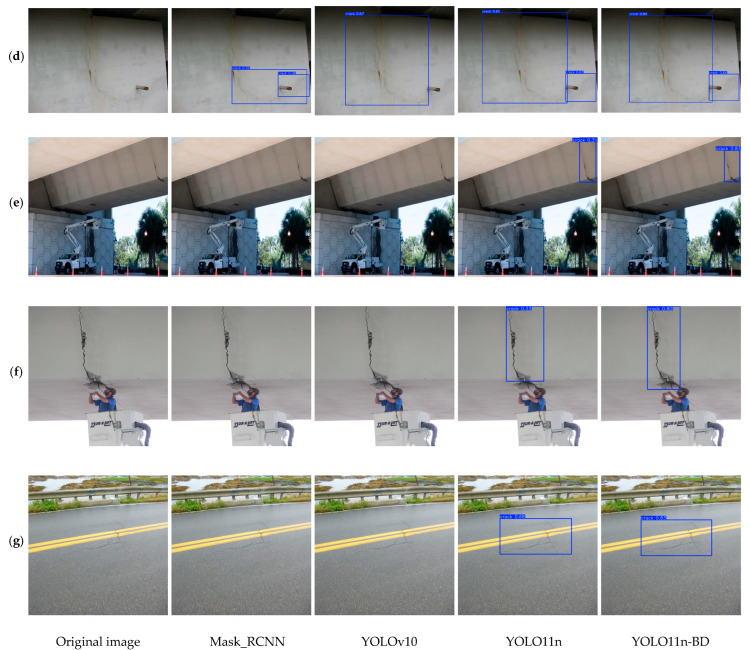
Comparison of bridge crack detection effects in complex backgrounds.

The trained object detection models were used to detect bridge crack images to evaluate further the application capability of the trained model YOLO11n-BD in real-world scenarios. This experiment aims to verify the model’s performance in complex backgrounds, particularly in cases where the bridge structure in the background may interfere with crack detection. It also aims to assess whether the model can still maintain high accuracy and robustness. The experiment selected and collected crack images with complex backgrounds as the test set. The test set used multiple resolution images to systematically evaluate the impact of input image resolution on algorithm performance, and then used Mask_RCNN, YOLOv10, YOLO11 and YOLO11-BD algorithms to detect them. The detection results are shown in Table 6 and Figure 11. Compared with other algorithms, YOLO11-BD demonstrates obvious advantages in both detection accuracy and computational efficiency, and it maintains high recognition accuracy even in complex scenarios involving bridge crack detection. YOLO11-BD performs significantly better in real-world scenarios than the other algorithms, particularly under complex background and lighting conditions. For example, in environments with many interfering objects (such as iron frames) and large background color differences, YOLO11-BD not only detects small cracks clearly but also provides precise bounding boxes. Moreover, as shown in Figure 11a–g, the YOLO11-BD algorithm can effectively avoid false detections and missed detections. Even in Figure 11c, only the YOLO11-BD algorithm can detect the crack. The experimental results indicate that YOLO11-BD performs excellently in high-noise backgrounds. Thus, it can effectively address the shortcomings of traditional models in complex backgrounds. It improves the accuracy and efficiency of crack detection, thereby demonstrating its high reliability and broad applicability in real-world detection and applications.

### 6.5. Activation Map Analysis

**Figure 12:**
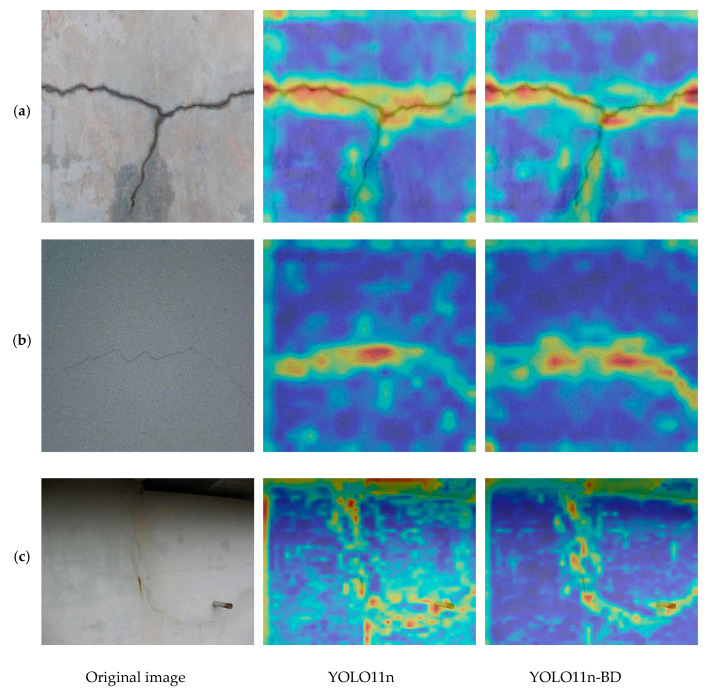
Comparison of activation maps with different algorithms.

To more intuitively validate the effectiveness of the improved algorithm module, this study analyzes the activation distribution changes in key feature maps between the YOLO11 model and the YOLO11-BD model, which incorporates the EMSCA and LDH modules. The activation maps are shown in Figure 12a–c. In the figure, red and yellow areas indicate stronger focus of the model, while the blue ones represent less focus. The original YOLO11 shows broader and more diffuse attention in the crack regions, leading to a reduction in accuracy. In contrast, YOLO11-BD maintains stronger focus even under noise, highlighting the potential of its improved attention mechanism in enhancing feature learning capability. The visualization of the activation maps demonstrates that the improved model exhibits more coherent and clearer boundary responses in complex backgrounds and fragmented crack regions, fully demonstrating its significant advantage in bridge crack detection.

## 7. Conclusions

Bridge cracks are an important indicator of bridge health and safety monitoring; thus, efficient, accurate and convenient crack detection has become a key research focus in bridge engineering. This study mainly aims to enhance the ability of real-time detection of bridge cracks using remote sensing devices, achieve high-accuracy detection in complex backgrounds and realize the lightweight design of the model. This study enhances the accuracy of bridge crack detection and achieves model lightweighting by improving the object detection algorithm YOLO11. An improved efficient multiscale conv module, namely EMSCA, is proposed to enhance the processing ability of feature maps. Thus, the detection accuracy can be improved, whilst maintaining computational efficiency. Additionally, lightweight detection head (LDH) with few parameters is introduced to further reduce the computational load of the model and improve the efficiency of real-time detection. The experimental results indicate that the YOLO11-BD algorithm proposed in this study achieves significant results in bridge crack detection, particularly in the significant effect of model lightweighting. Compared with the original YOLO11 network, YOLO11-BD exhibits improved mAP50 and mAP50-95 by 3.1% and 4.8%, respectively, whereas its GFLOPs is decreased by 19.05%. As a result, the computational load is significantly reduced, and the detection efficiency is improved. FPS is also maintained at a high level. This study helps to deploy the model on various remote sensing devices to achieve convenient and efficient detection of bridge cracks in different environments and provide important technical support for bridge health monitoring. In the future, the algorithm model developed in this study is planned to be deployed on various types of mobile devices with different performance capabilities, and experiments will be conducted in various real and complex scenarios to test and optimize the model’s performance further.

## Figures and Tables

**Table 1:** Comparison of different YOLO algorithms.

Model	Anchor	Input	Backbone	Neck
YOLOv8	No	640 × 640 × 3	CBS + C2f + SPPF	SPP/PAN
YOLOv9	Yes	640 × 640 × 3	CBS + G-ELAN	PAN
YOLOv10	No	640 × 640 × 3	PSA	C2f/PAN
YOLO11	No	640 × 640 × 3	CBS + C3k2 + SPPF + C2PSA	C3k2/PAN

**Table 2:** Results of the ablation experiment.

YOLO11n	EMSCA	LDH	F1/%	mAP50/%	mAP50-95/%	GFLOPs	FPS
√			86.8	91.5	68.1	6.3	526
√	√		89.4	94.0	71.3	6.3	555
√		√	88.4	94.1	70.7	5.1	526
√	√	√	89.2	94.3	71.4	5.1	555

‘√’ indicates the model or module used in the experiment.

**Table 3:** Comparison of detection performance with different algorithms.

Algorithm	F1/%	mAP50/%	mAP50-95/%	GFLOPs	FPS
Mask-RCNN	77.4	85.2	60.3	133.6	138
YOLOv10	80.5	89.0	66.4	8.2	500
YOLO11	86.8	91.5	68.1	6.3	526
YOLO11-BD	89.2	94.3	71.4	5.1	555

**Table 4:** Training effects of different algorithms in RTX 4060 environment.

Algorithm	F1/%	mAP50/%	mAP50-95/%	GFLOPs	FPS
YOLO11	88.8	92.1	70.6	6.3	263
YOLO11-BD	89.5	94.6	73.3	5.1	333

**Table 5:** The results of the five-fold cross-validation.

Fold	Training Set	Validation Set	F1/%	mAP50/%	mAP50-95/%
1	Fold 2, 3, 4, 5	Fold 1	89.2	94.4	71.5
2	Fold 1, 3, 4, 5	Fold 2	89.3	94.7	71.9
3	Fold 1, 2, 4, 5	Fold 3	89.1	94.2	71.1
4	Fold 1, 2, 3, 5	Fold 4	89.4	95.1	72.4
5	Fold 1, 2, 3, 4	Fold 5	88.9	93.9	71.3
Average			89.2	94.5	71.6

**Table 6:** Evaluation results on the test set in complex scenarios.

Algorithm	F1/%	mAP50/%	mAP50-95/%	GFLOPs
Mask-RCNN	55.3	60.2	41.4	133.6
YOLOv10	60.7	68.5	47.4	8.2
YOLO11	74.9	77.8	54.7	6.3
YOLO11-BD	78.5	83.6	57.1	5.1

## Data Availability

The dataset can be found at https://universe.roboflow.com/yuanjiashuo/bridge-crack-hlaig, uploaded on 9 May 2025.
